# A TaqMan-Based qRT-PCR Assay for Accurate Evaluation of the Oncogenic *TrkAIII* Splice Variant in Tumor cDNAs

**DOI:** 10.3390/cancers17030471

**Published:** 2025-01-30

**Authors:** Maddalena Sbaffone, Antonietta Rosella Farina, Ilaria Martelli, Eugenio Pontieri, Stefano Guadagni, Andrew Reay Mackay, Lucia Cappabianca, Veronica Zelli

**Affiliations:** Department of Biotechnological and Applied Clinical Sciences, University of L’Aquila, Via Vetoio, 67100 L’Aquila, Italy; maddalena.sbaffone@graduate.univaq.it (M.S.); antonietta.farina@univaq.it (A.R.F.); ilaria.martelli@graduate.univaq.it (I.M.); eugenio.pontieri@univaq.it (E.P.); stefano.guadagni@univaq.it (S.G.); luciaannamaria.cappabianca@univaq.it (L.C.); veronica.zelli@univaq.it (V.Z.)

**Keywords:** NTRK1/TrkA, alternative TrkAIII splicing, TaqMan-based qRT-PCR, tumor cDNAs, precision oncology, Trk inhibitor therapy

## Abstract

**Background:** Alternative *NTRK1/TrkA* splicing resulting in *TrkAIII* expression, originally discovered in advanced-stage metastatic neuroblastomas, is also pronounced in prostate, medullary thyroid, glioblastoma multiforme, MCPyV-positive Merkel cell, cutaneous malignant melanoma, and pituitary neuroendocrine tumor subsets. In tumor models, TrkAIII exhibits actionable oncogenic activity equivalent to the *TrkT3*-fused oncogene, and in tumor cell lines, alternative *TrkAIII* splicing is promoted by hypoxia, nutrient deprivation, endoplasmic reticulum stress, and SV40 large T antigen, implicating tumor microenvironmental conditions and oncogenic polyoma viruses in tumor-associated TrkAIII expression. Collectively, these observations characterize TrkAIII as a potentially frequent, actionable oncogenic alternative to *TrkA* gene fusion in different tumor types. Currently, therapeutic approval for efficacious Trk inhibitors is restricted to *Trk-fused* gene positive tumors and not for tumors potentially driven by TrkAIII. **Methods:** With the therapeutically relevant aim of improving the identification of tumors potentially driven by TrkAIII, we have developed a TaqMan-based qRT-PCR assay for evaluating *TrkAIII* expression in tumor cDNAs. **Results:** This assay, validated using gel-purified *fs-TrkA* and *TrkAIII* cDNAs alone and in complex cDNA mixtures, employs primers and probes designed from *fs-TrkA* and *TrkAIII* sequences, with specificity provided by a TaqMan probe spanning the *TrkAIII* exon 5–8 splice junction. It is highly efficient, reproducible, and specific and can detect as few as 10 *TrkAIII* copies in complex RNAs extracted from either fresh or FFPE tumor tissues. **Conclusions:** Inclusion of this assay into precision oncology algorithms, when paired with *fs*-TrkA qRT-PCR and TrkA immune histochemistry, will make it easier to identify patients with therapy-resistant, advanced-stage metastatic *Trk-fused* gene-negative tumors potentially driven by TrkAIII, for whom approval of third-line effective Trk inhibitors could be extended.

## Simple Summary

We have developed and validated a novel TaqMan-based qRT-PCR assay to detect and quantify expression of the oncogenic *NTRK1/TrkA* splice variant *TrkAIII* in tumor tissues. This assay was developed based on the discovery that TrkAIII is a potentially frequent and actionable oncogenic equivalent to *TrkA-fused* genes in neuroblastomas and other tumor types and is highly efficient, reproducible, and specific in detecting as few as 10 *TrkAIII* copies in complex tumor cDNAs. Inclusion of this assay into precision oncology algorithms will make it easier to identify patients with therapy-resistant, advanced stage, metastatic *Trk-fused* gene negative tumors potentially driven by TrkAIII, for whom approval of third-line effective Trk inhibitors could be extended.

## 1. Introduction

The discovery and characterization of human *Trk* and *TrkT1-3* as novel fused oncogenes containing the same tyrosine kinase domain in colon and papillary thyroid tumors [1,2] preceded and facilitated the identification of the *NTRK1/TrkA* gene (NG_007493.1) [3], which encodes tropomyosin-related kinase A (TrkA), the high-affinity tyrosine kinase receptor for the neurotrophin nerve growth factor, which is localized on chromosome 1p23.1 and is composed of 17 exons [4,5,6,7]. Additional *TrkA-fused* oncogenes have since been identified, with low frequencies in many common adult cancers and with higher frequencies in some rare tumor types, including secretory breast carcinoma, mammary analogue secretory carcinoma, and congenital infantile fibrosarcoma [8,9]. Subsequent development of small-molecule Trk inhibitors has led to breakthrough clinical studies in *Trk-fused* gene-positive pediatric and adult cancers, which have reported profound durable responses to the Trk inhibitors larotrectinib and entrectinib [10,11]. These studies accelerated the clinical approval of both drugs for the treatment of therapy-resistant, advanced-stage, metastatic pediatric and adult *Trk-fused* gene-positive cancers, and these drugs continue to elicit profound durable responses (see clinical trials NCT05192642, NCT02650401, NCT04589845, and NCT02568267) [8,9,10,11,12,13]. However, these inhibitors are not currently FDA approved for the treatment of *Trk-fused* gene-negative tumors driven by alternative oncogenic *NTRK1/TrkA* activation mechanisms [14,15].

Alternative *NTRK1/TrkA* splicing, resulting in expression of the *TrkAIII* splice variant (GeneBank OP866787), represents a potential oncogenic alternative to *Trk-fused* genes in different tumor types [16,17,18,19,20,21,22,23,24]. Alternative TrkAIII splicing was originally discovered in human neuroblastomas (NBs) [16], and it has been reported to significantly correlate with advanced-stage metastatic disease and post-therapeutic relapse [16,17]. *TrkAIII* mRNA exhibits cassette exons 6–7 and exon 9 skipping and is expressed as an in-frame receptor variant characterized by the omission of the extracellular D4 domain and associated N-glycosylation sites cell, required for surface expression and the prevention of the ligand-independent activation of fully spliced TrkA [18,19]. As a consequence, TrkAIII is not expressed at the cell surface but re-localizes to pre-Golgi membranes, centrosomes, and mitochondria, where it exhibits a ligand-independent cell cycle and stress-regulated activation, resulting in oncogenic activity [16,20,21,22,23,24]. In tumor models, TrkAIII transforms NIH-3T3 cells and promotes primary and metastatic tumorigenicity in nude mouse NB models through mechanisms that include pro-survival and pro-angiogenic PI3K/Akt signaling; increased SOD2 and stem cell-associated gene expression; centrosome amplification, leading to increased chromosome instability; stress-induced metabolic glycolytic adaptation; and enhanced mitochondrial protection against deregulated Ca^2+^ homeostasis [16,20,21,22,23,24]. In these models, TrkAIII also exhibits oncogenic equivalence to the *TrkA-fused* oncogene *TrkT3* and is inhibited by approved entrectinib and lestaurtinib Trk inhibitors [16,20,21,22,23,24]. Collectively, these observations characterize TrkAIII as an actionable pathological equivalent to *TrkA-fused* oncogenes in addition to being the pathological version of a previously described engineered D4 domain-deleted TrkA oncogene [16,17].

TrkAIII involvement in tumors other than NB is supported by reports of pronounced alternative *TrkAIII* splicing and exclusive *TrkAIII* mRNA expression in subsets of prostate carcinoma (PRC), medullary thyroid carcinoma (MTC) [16], glioblastoma multiforme (GBM) [21], MCPyV-positive Merkel cell carcinoma (MCC), cutaneous malignant melanoma (CMM), and pituitary neuroendocrine tumors (PitNET), and of frequent association with evidence of intracellular TrkAIII activation in MCPyV MCCs, CMMs, and PitNETs [25,26,27,28]. The actionable potential of TrkAIII, confirmed by its inhibition by approved Trk inhibitors [16,20,23,24,27], is also supported by the promotion of tumor cell alternative *TrkAIII* splicing by conditions that characterize tumor microenvironments (TMEs) (hypoxia, nutrient deprivation, and ER stress response activation) and by SV40 polyomavirus large T antigen, implicating oncogenic poliomaviruses in promoting sustained alternative *TrkAIII* splicing in tumors and providing a potential explanation for the pronounced alternative *TrkA* splicing detected in MCPyV-positive (but not -negative) MCCs [25,26]. Furthermore, a remarkable and durable response to entrectinib Alk-Trk inhibitory therapy has been reported in an infant with therapy-resistant, advanced stage 4 metastatic NB exhibiting a molecular profile consistent with TrkAIII expression and activation [29]. Moreover, sequence analyses have so far identified *TrkAIII* mRNA as the only *NTRK1/TrkA* splice variant in tumor samples with an in-frame tyrosine kinase domain and, therefore, oncogenic tyrosine kinase potential [16,20,25,26,27,28], which reinforces this hypothesis. Collectively, these observations support a potentially frequent and actionable role for alternative *TrkAIII* splicing as an oncogenic alternative to *TrkA-gene* fusion in different tumor types, consistent with a report that alternative splicing is a significantly frequent oncogene activation mechanism in a wide variety of tumor types [30] and in particular tumors with low mutation burdens, such as NBs, MCPyV-positive MCCs, PRCs, GBMs, and MTCs [30,31,32].

Trk inhibitors, which work at the protein level, are currently FDA approved for third-line treatment of therapy-resistant, advanced-stage, metastatic *Trk-fused gene*-positive tumors [14,15]. These tumors are identified by precision oncology algorithms that include next-generation sequencing, fluorescent in situ hybridization, qRT-PCR and immunohistochemistry [33,34]. Considering that TrkAIII is a potentially actionable *TrkA-fused* oncogene equivalent and that alternative *TrkAIII* splicing is pronounced and occasionally exclusive in advanced-stage, metastatic and relapsed NBs, PRCs, MTC, GBs, MCCs, CMMs, and PitNET tumors [16,17,20,25,26,27,28], and considering the current lack of a *TrkAIII*-specific real-time qPCR detection method, we have developed a sensitive *TrkAIII*-specific TaqMan-based qRT-PCR assay for quantifying *TrkAIII* expression in complex tumor cDNAs. The inclusion of this assay into precision oncology algorithms will make it easier to identify patients with *Trk-fused* gene-negative tumors, potentially driven by TrkAIII, for whom approval of third-line effective Trk inhibitors could be extended.

## 2. Materials and Methods

### 2.1. fs-TrkA and TrkAIII cDNA Purification and Sequencing for TaqMan qRT-PCR Validation

**Figure 1:**
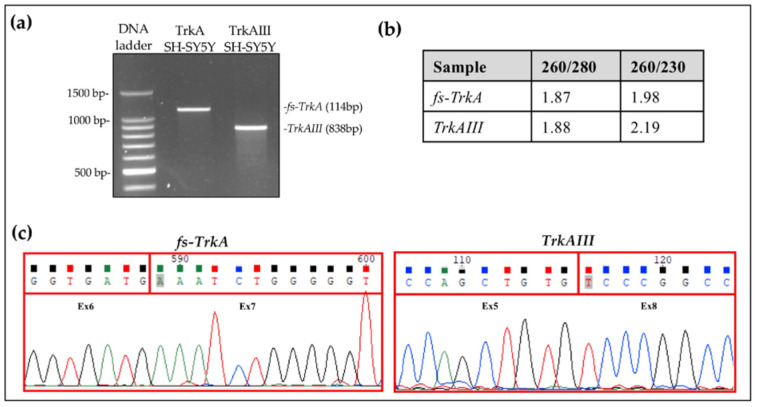
(**a**) Agarose gel of purified *fs-TrkA* and *TrkAIII* fragments generated using primers spanning *TrkA* exons 1–8. (**b**) Fragment quality assessed by Nanodrop spectrophotometry. (**c**) Sequences of purified *fs-TrkA* exon 6–7 and *TrkAIII* exon 5–8 splice junctions.

Fully spliced (*fs-*) *TrkA* and alternatively spliced *TrkAIII* cDNAs from previously characterized stable transfected TrkA and TrkAIII SH-SY5Y cell lines [16] were PCR amplified using the primer sets 5′-ATGCTGCGAGGCGGACGGCGC-3′ and 5′-GGAGGCCTGGCCGAAGGGGTT-3′, separated by 1.5% agarose gel electrophoresis, and purified from gel slices using the ReliaPrep™ DNA Clean-Up and Concentration System, as described by the manufacturer (Promega, Madison, WI, USA). PCR product quantity and purity were then determined by Nanodrop spectrophotometry, as directed by the manufacturer (Thermo Fisher Scientific, Waltham, MA, USA), and the region of interest for *TrkAIII*-specific qRT-PCR reactions was confirmed to be non-mutated by double-stranded Sanger sequencing using BigDye Terminator V.2.1. Cycle Sequencing kit in an ABI PRISM 310 Genetic Analyzer, as described by the manufacturer (Thermo Fisher Scientific, Waltham, MA, USA) (Figure 1) (sequence data was generated using ABI Prism GeneScan Analysis 3500 Series Data Collect software version 3.1). The Sanger sequence and Blast of the *TrkAIII* cDNA region corresponding to the Assay 1 qRT-PCR amplicon are presented in Supplementary Figure S1.

### 2.2. Cell Lines, Tumor Tissues, RNA Purification, and Reverse Transcription Reactions

IMR32 [CCL-127] and HEPG2 (HB-8065) cell lines were obtained from ATCC (Manassas, VI, USA); the NB1 cell line (CVCL_1440) was obtained from Riken BRC (Tsukuba, Japan); and the stable transfected pcDNA, TrkA, and TrkAIII SH-SY5Y cell lines have been previously described [16]. Cell lines were routinely cultured at 37 °C in a 5% CO_2_ atmosphere, in either RPMI 1640 or DMEM medium (Euroclone, Milan, Italy) supplemented with 10% fetal bovine serum, 1% glutamine, and 1% penicillin/streptomycin (Euroclone, Milan, Italy). Stable pcDNA, TrkA, and TrkAIII SH-SY5Y transfectants were also cultured with zeocin (200 μg/mL) (Thermo Fisher Scientific, Waltham, MA, USA). *Saccharomyces boulardii* yeast cells were cultured to stationary phase in YPD broth containing 1% *w*/*v* yeast extract, 2% *w*/*v* peptone, and 2% *w*/*v* D-glucose (Biolife Italiana, Milan, Italy) at 30 °C in a horizontal shaking incubator. The fresh and formalin-fixed paraffin-embedded (FFPE) human CMM tissues used in this study have been previously described [27] and are presented in Supplementary Table S1.

For RNA extractions, fresh tumor surgical specimens were placed in RNAse-free tubes, snap-frozen in liquid nitrogen, and stored at −80 °C until used. Tumor regions were selected macroscopically and macro-dissected into 3 mm^2^ pieces in order to minimize non-tumor tissue contamination. Tissues were then pulverized in a Tissue Lyser LT (Quiagen, Milan, Italy), at 5 oscillations per second for 2 min, lysed in Quick-RNA^TM^ Miniprep Kit lysis buffer and RNAs purified from lysates, as directed by the manufacturer (Zymo Research, Freiberg im Breisgau, Germany). For FFPE tumor samples, RNAs were purified from 50 μm FFPE sections using PureLink^TM^ FFPE RNA isolation kit as directed by the manufacturer (Thermo Fisher Scientific, Waltham, MA, USA). For tumor cell lines and *Saccharomyces boulardii* yeast, cells were lysed in Quick-RNA^TM^ Miniprep kit lysis buffer and RNAs purified from lysates as directed by the manufacturer (Zymo Research, Freiberg im Breisgau, Germany). RNA purity and concentration were evaluated by Nanodrop spectrophotometry, as directed by the manufacturer (Thermo Fisher Scientific, Waltham, MA, USA). Purified RNAs (500 ng) exhibiting spectrophotometer 260/280 values > 1.9 were then reverse transcribed using a Superscript IV VILO MasterMix reverse transcription kit as directed by the manufacturer (Thermo Fischer Scientific, Waltham, MA, USA).

### 2.3. Assay Design

**Figure 2:**
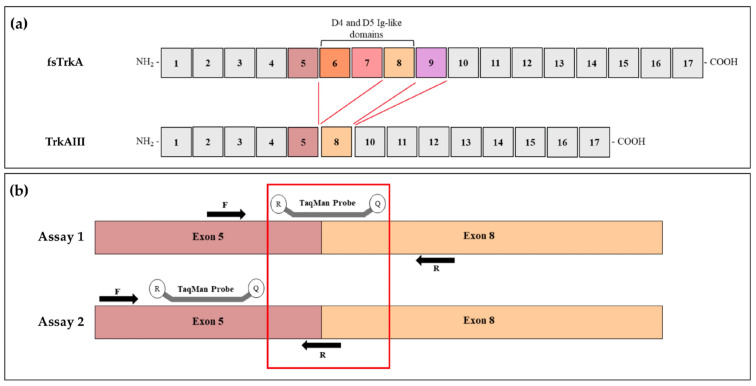
(**a**) Comparative representation of *TrkAIII* and *fs-TrkA* exon structures. (**b**) TrkAIII qRT-PCR Assay 1 and 2 strategies, illustrating *TrkAIII* specificity provided by the TaqMan probe and by the antisense primer (R), respectively.

Assay primers and probes were selected from *NTRK1/TrkA* (NM_001012331.1) and *TrkAIII* (GenBank: OP866787.1) sequences using Custom TaqMan^®^ Assay Design Tool software (https://www.thermofisher.com/order/custom-genomic-products/tools/genotyping/, accessed on 7 March 2023) and were synthesized by Thermo Fisher Scientific (Waltham, MA, USA). A 473 base pair (bp) region spanning the *TrkAIII* exon 5–8 junction was selected as the target sequence for primer and probe design, and 2 independent qRT-PCR assays (Assay 1 and Assay 2) were evaluated utilizing TaqMan^TM^ probes with 5′ 6-carboxyfluoroscein dye labels and 3′ minor groove binder non-fluorescent quenchers. For Assay 1 (cat. number 4331348, assay ID APRWNPP), the forward *NTRK1/TrkA* exon 5 primer (5′-GCCCACATGCCCAATGC-3′) and reverse *NTRK1/TrkA* exon 8 primer (5′-CACCGCCGTGTGCAG-3′) were designed to amplify a 57 bp product to be used in conjunction with a TaqMan probe (5′-CTGTGTCCCGGCCAGTGT-3′) spanning the *TrkAIII* exon 5–8 splice junction. For Assay 2 (cat. number 4331348, assay ID AP2XGU9), the forward *NTRK1/TrkA* exon 5 primer (5′-GAACAGAAGCTGCAGTGTCATG-3′) and reverse *TrkAIII* exon 5–8 splice junction primer (5′-CACTGGCCGGGACACA-3′) were designed to amplify a 73 bp product to be used in conjunction with the TaqMan probe (5′-CACATGCCCAATGCCA-3′) localized within *NTRK1/TrkA* exon 5 sequence (Figure 2). Therefore, *TrkAIII* specificity in Assay 1 was determined by the probe and in Assay 2 by the antisense primer.

### 2.4. Real-Time qPCR Analysis

Quantitative (q)RT-PCR analyses were performed in a final volume of 20 μL containing 10 μL of TaqMan Universal Master Mix II (2×) without uracil-N-glycosylase (TaqMan Universal Master Mix II, No UNG. Thermo Fisher Scientific, Waltham, MA, USA), 1 μL of Custom TaqMan Gene Expression Assay (20×), and 1 μL of sample template and nuclease-free water to adjust reaction volumes. Reactions were then amplified in a 7500 Fast Real-Time PCR System (Thermo Fisher Scientific, Waltham, MA, USA) as follows: 10 min incubation at 95 °C followed by 40 cycles of 15 s at 95 °C and 1 min at 60 °C. All reactions were performed in triplicate, and a negative control was included in each experiment. In selected cell lines and tumor samples, *β-actin* expression was also evaluated in parallel to *TrkAIII,* under identical PCR conditions, using the Human ACTB (*β-actin*) Endogenous Control Assay, as directed by the manufacturer (Hs01060665_g1, Thermo Fisher Scientific, Waltham, MA, USA).

Analytical performance parameters were assessed using 7 different concentrations of purified *TrkAIII* PCR fragments ranging from 10 to 10^7^ copies alone or added to 25 ng of *TrkAIII*-negative *Saccharomyces boulardii* yeast cDNAs, and for confirmation of specificity, capacity to amplify and detect 10^7^ and 10^6^ copies of either purified *fs-TrkA* or *TrkAIII* PCR fragments were compared. The number of copies per μL (C) was calculated using the following equation: C = PA ÷ GMX (where P = PCR product concentration (ng/μL); A = Avogadro′s constant (6 × 10^23^); G = the amplicon size in bp; M = multiplying constant (1 × 10^9^), and X = the average molecular weight of 1 bp in the amplicon (650 Daltons)). Amplification efficiency was also evaluated in 5 different concentrations of TrkAIII SH-SY5Y cell cDNA ranging from 0.0005 to 5 ng. Specificity was determined by detection of *TrkAIII* but not *fs-TrkA* cDNA.

### 2.5. Real-Time PCR Analysis of Cell Line and Tumor Tissue cDNAs

*TrkAIII* copy numbers were evaluated in complex cDNAs, including cDNAs from cell lines and fresh and FFPE tumor tissues and confirmed to express different levels of *TrkAIII* by semi-quantitative RT-PCR [25,27]. Briefly, experiments contained 1–3 μL of sample template (25 ng of cell line and fresh tumor tissue cDNAs or 70 ng of FFPE tissue cDNAs), and reactions were performed as described above. Absolute quantification of *TrkAIII* copy numbers was deduced from standard curves prepared using serial dilutions of the purified *TrkAIII* cDNA fragment.

Accompanying semi-quantitative RT-PCR analyses of cell line and fresh tumor tissue cDNAs utilized the primer set 5′-AGAAGCTGCAGTGTCATGGG-3′ and 5′-ATTGAGCACGGAGCCATTGA-3′; consisted of 35 cycles of 40 s denaturation at 95 °C, 30 s annealing at 58 °C, and 40 s of elongation at 72 °C; and generated a 452 bp *fs-TrkA* amplicon and a 176 bp *TrkAIII* amplicon. In order to mitigate fixative-dependent RNA degradation [35], semi-quantitative RT-PCR of FFPE tumor cDNAs used the primer set 5′-AATGCCAGCTGTGTCCCG-3′ and 5′-TGGTCTCATTGAGCACGGAG-3′ and consisted of 40 cycles of 30 s denaturation at 95 °C, 30 s of annealing at 60 °C, and 30 s of elongation at 72 °C and generated a 139 bp *TrkAIII* amplicon. As a general rule, annealing temperatures for semi-quantitative PCR and RT-PCR reactions were set 2 °C lower than the lowest melting temperature of the two oligonucleotide primers used, provided by the manufacturer (Integrated DNA Technologies, TEMA Ricerca, Bologna Italy). Semi-quantitative RT-PCR products were separated by 1.5% agarose gel electrophoresis and digitally photographed in a ChemiDoc MP Basic Imaging System (Bio-Rad, Hercules, CA, USA).

### 2.6. Data Analysis

TaqMan qRT-PCR data were captured and analyzed by 7500 Software version 2.3 (Thermo Fisher Scientific, Waltham, MA, USA), and the following parameters were evaluated: (i) Amplification efficiency (optimal values: 100 ± 10% with correlation coefficient R^2^ values > 0.985), determined by 7500 Software version 2.3, was calculated from the formula E = (10^−1/slope^) − 1, where E is presented as a percentage (slope values close to −3.32 indicate optimal, close to 100%, amplification efficiency, and they may exceed 100%). Amplification efficiency values between 90 and 105% indicate high efficiency. Regression coefficient R^2^ values, also determined by 7500 Software version 2.3, indicate the closeness of fit between the standard curve regression line and the individual standard curve Ct data points, and should be > 0.985 (R^2^ values of 1.00 indicate a perfect fit) [36]; (ii) sensitivity (minimum number of copies detectable); (iii) specificity for *TrkAIII* but not *fs-TrkA*; and (iv) reproducibility of mean cycle threshold (Ct) values, standard deviation (SD), and coefficients of variation (CVs) in 3 experiments, each performed in triplicate under identical conditions. Graphical illustrations were prepared using GraphPad Prism 10 software version 10.1.1 (GraphPad Software Inc., San Diego, CA, USA). Relative quantification of *TrkAIII* expression in selected cell lines and tumor samples were determined by the Ct normalization algorithm [37] using *β-actin* expression as the endogenous control.

## 3. Results

### 3.1. Evaluation of TrkAIII Real-Time PCR Assays Analytical Performance

**Figure 3:**
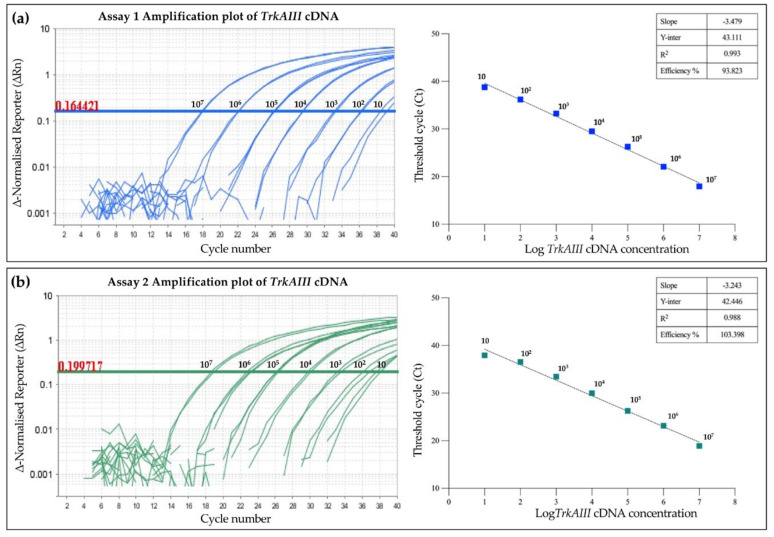
Efficiency and sensitivity of (**a**) Assay 1 and (**b**) Assay 2 in detecting different concentrations of purified TrkAIII cDNA fragments ranging from 10 to 10^7^ copies, displayed as amplification plots (left panels, with threshold values indicated in red), a line graph of Ct values (right panels), and tables displaying slope, Y-intercept, R^2^, and efficiency (%) values.

Purified *TrkAIII* PCR fragments (Figure 1), containing target regions for primer and probe sets and at concentrations ranging from 10 to 10^7^ copies, were subjected to TaqMan qRT-PCR in order to produce standard curves (Figure 3).

In these reactions, Assay 1 efficiency was calculated to be 93.823% (slope = −3.479 and R^2^ = 0.993) (Figure 3a), and Assay 2 efficiency was calculated to be 103.398% (slope = −3.243, R^2^ = 0.988) (Figure 3b). Both assays detected as few as 10 TrkAIII copies, confirming high sensitivity.

**Figure 4:**
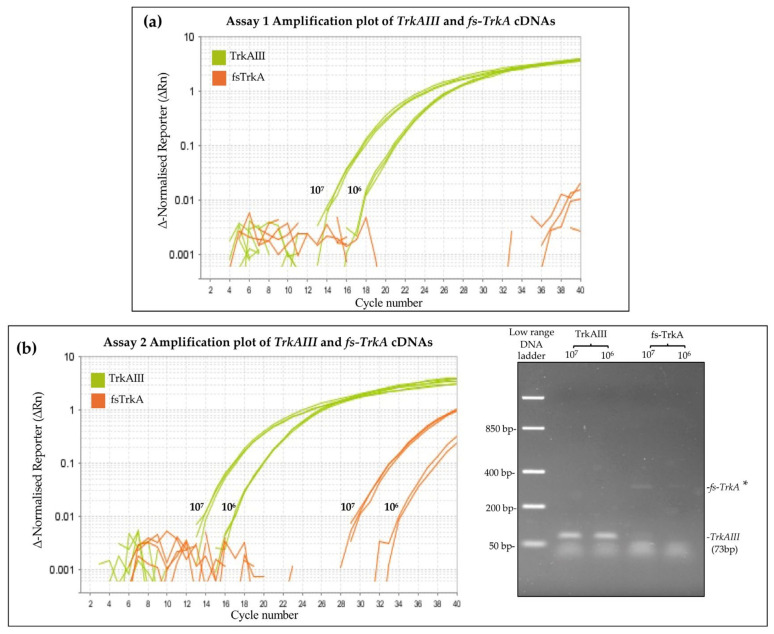
Comparison of Assay 1 and Assay 2 specificity in detecting *TrkAIII* but not *fs-TrkA cDNA*. Amplification plots demonstrating (**a**) *TrkAIII*-specific qRT-PCR signals from *TrkAIII* but not *fs-TrkA* cDNAs in Assay 1, and (**b**) qRT-PCR signals for both *TrkAIII* and *fs-TrkA* plus an agarose gel demonstrating both expected *TrkAIII* and unexpected (*) *fs-TrkA* amplification in Assay 2.

With respect to specificity, Assay 1 and Assay 2 were compared in their capacity to specifically detect 10^7^ and 10^6^ copies of purified *TrkAIII* but not 10^7^ and 10^6^ copies of purified *fs-TrkA* PCR fragments. In Assay 1 (specificity provided by the TaqMan probe), both *TrkAIII* and *fs-TrkA* cDNAs were amplified, but only the *TrkAIII* product was detected by the TaqMan probe. In Assay 2 (specificity provided by the reverse primer), significant unexpected *fs-TrkA* amplification occurred and was detected by the TaqMan probe. These data confirm the optimum specificity for Assay 1 but not Assay 2 (Figure 4).

In Assay 1, intra-assay and inter-assay reproducibility was determined in three independent experiments under identical conditions, each performed in triplicate. These experiments produced coefficients of variation (CVs) from Ct values of <0.005 and <0.04, respectively (Table 1). The calculated efficiencies of the three independent experiments were 93.823% (R^2^ = 0.993, slope −3.479) (Figure 3), 94.773% (R^2^ = 0.992, slope −3.454), and 90.475% (R^2^ = 0.995, slope −3.573), resulting in a mean (±s.e.) calculated efficiency of 93.024 (±1.303)%. As qRT-PCR CV values < 0.1 indicate low variability [38], the data confirm optimal reproducibility and reliability in detecting and quantifying *TrkAIII*.

**Figure 5:**
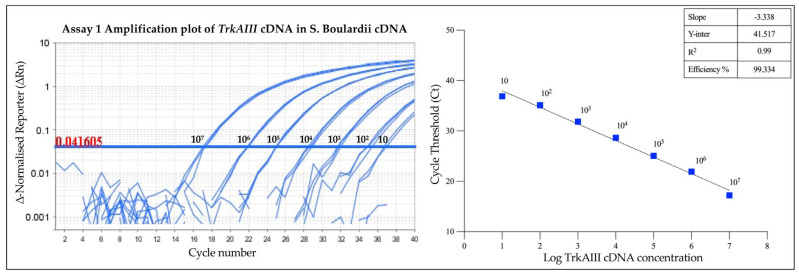
Evaluation of Assay 1’s efficiency and sensitivity in detecting different concentrations of purified *TrkAIII* cDNA fragments, ranging from 10 to 10^7^ copies added to 25 ng of *TrkAIII*-free *Saccharomyces boulardii* cDNA, in one of the two independent experiments, displayed as amplification plots (left panel, with the threshold value indicated in red), a line graph of Ct values (right panel), and a table displaying slope, Y-intercept, R^2^, and efficiency (%) values.

Assay 1’s efficiency and sensitivity were subsequently investigated in complex *TrkAIII*-negative *Saccharomyces boulardii* cDNA spiked with different concentrations of purified TrkAIII fragments ranging from 10 to 10^7^ copies. The calculated efficiencies of the two independent experiments were 99.334% (R^2^ = 0.99, slope −3.338) (Figure 5) and 90.427% (R^2^ = 0.993, slope −3.575) for the duplicate experiment, resulting in a mean (±s.e.) calculated efficiency of 94.88 (±3.363)%. These data confirm that cDNA complexity does not interfere with either the efficiency or sensitivity of *TrkAIII* detection (Figure 5).

Assay 1’s intra-assay and inter-assay reproducibility in detecting *TrkAIII* cDNA in TrkAIII-free *Saccharomyces boulardii* cDNA was determined in duplicate independent experiments, each performed in triplicate under identical conditions. These experiments produced coefficients of variation (CVs) from Ct values of <0.01 and <0.028, respectively, confirming optimal reproducibility and reliability in detecting and quantifying *TrkAIII* in complex cDNAs (Table 2).

**Figure 6:**
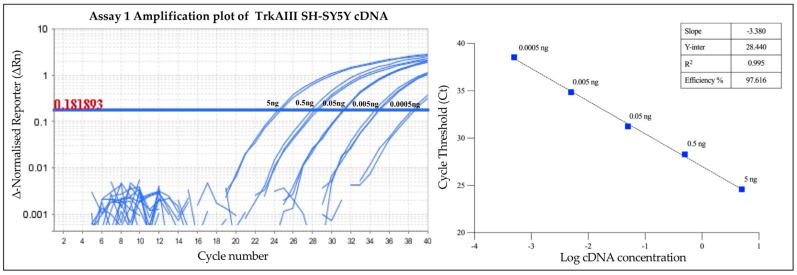
Assay 1 efficiency and sensitivity in detecting *TrkAIII* in different concentrations of TrkAIII SH-SY5Y cDNAs ranging from 0.0005 to 5 ng in one of the two independent experiments, displayed as amplification plots (left panel, with threshold value indicated in red), a line graph of Ct values (right panel), and a table displaying slope, Y-intercept, R^2^, and efficiency (%) values.

In addition, Assay 1’s efficiency was also evaluated in different concentrations of stable transfected TrkAIII SH-SY5Y cDNA, ranging from 0.0005 to 5 ng. The calculated efficiencies of the two independent experiments were 97.616% (R^2^ = 0.995, slope −3.380) (Figure 6) and 90.609% (R^2^ = 0.986, slope −3.570) for the duplicate experiment, resulting in a mean (±s.e.) calculated efficiency of 94.112 (±2.860)%. These data further confirm the optimum Assay 1 efficiency in detecting *TrkAIII* in complex cell line cDNAs.

Assay 1’s intra-assay and inter-assay reproducibility in detecting *TrkAIII* in different concentrations of TrkAIII SH-SY5Y cDNAs was determined in independent experiments, each performed in triplicate under identical conditions. These experiments produced coefficients of variation (CVs) from Ct values of <0.013 and <0.039, respectively, further confirming optimal reproducibility and reliability in detecting and quantifying *TrkAIII* in complex cDNAs (Table 3) [36].

### 3.2. Detection of TrkAIII in Complex Tumor Cell Lines and in Fresh and FFPE Tumor cDNAs

**Figure 7:**
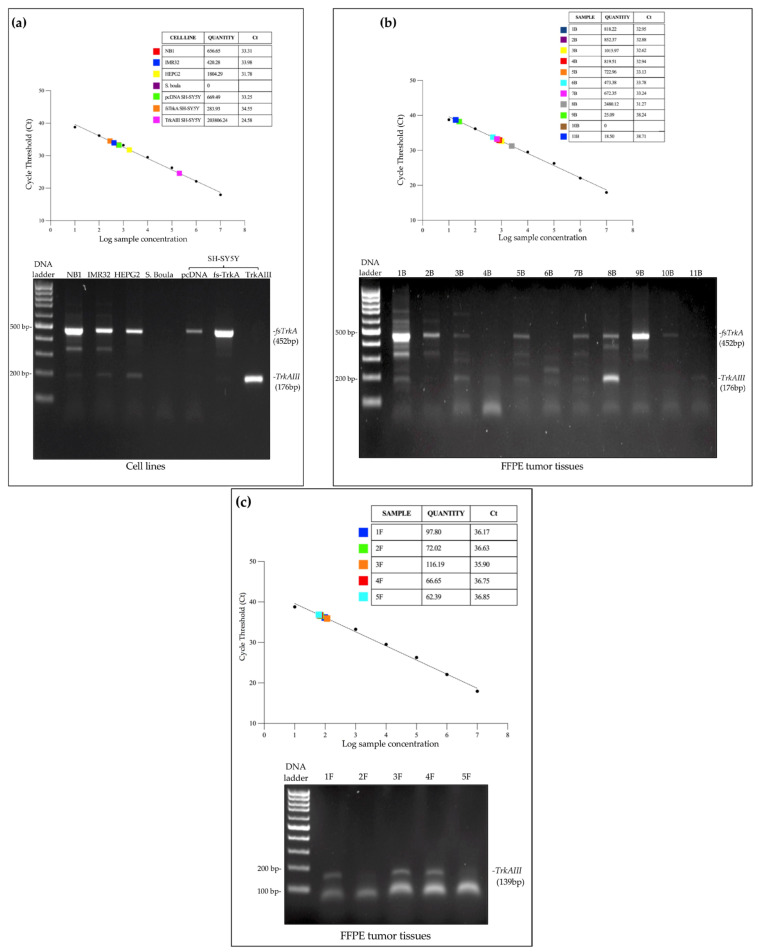
Diagnostic potential of Assay 1 in absolute quantification of *TrkAIII* mRNA levels in cDNAs from (**a**) NB1, IMR32, HEPG2, *Saccharomyces boulardii* (S. boula), pcDNA SH-SY5Y, *fs*-TrkA SH-SY5Y, and TrkAIII SH-SY5Y cell lines; (**b**) cDNAs from 11 fresh CMM tissues (1–11B); and (**c**) cDNAs from 5 FFPE CMM tissues (1–5F), plus agarose gel images demonstrating 452 bp *fs-TrkA* and 176 bp *TrkAIII* products amplified by semi-quantitative PCR in cell lines and fresh tumor cDNAs and 139 bp product amplified by semi-quantitative PCR in FFPE tumor cDNAs using the primer sets reported in Section 2.5.

Assay 1 was subsequently evaluated in its ability to detect *TrkAIII* expression in complex cDNAs from tumor cell lines (n = 6), fresh tumor tissues (n = 11), and FFPE tumor tissues (n = 5) in comparison with semi-quantitative RT-PCR. In terms of absolute quantification and signal intensity, Assay 1 detected *TrkAIII* expression in cell lines, in 10 of 11 fresh tumor samples (not detected in 10B), in all FFPE tumor tissue samples, and in all samples positive for TrkAIII by semi-quantitative RT-PCR (Figure 7). Absolute *TrkAIII* copy numbers in 25 ng of cell line cDNAs, calculated from Ct standard curves, were ≈657 in NB1 cDNA, ≈420 in IMR32 cDNA, and ≈1805 in 25 ng of HEPG2 cDNA, and in the SH-SY5Y transfectants they were ≈670 in pcDNA SH-SY5Y cDNA, ≈284 in *fs-*TrkA SH-SY5Y, and ≈203,807 in TrkAIII SH-SY5Y cDNA (Figure 7a). In 25 ng of fresh tumor cDNAs, *TrkAIII* copy numbers ranged from ≈19 in sample 11B to 2480 in sample 8B, and *TrkAIII* was not detected in sample 10B (Figure 7b). In 70 ng of FFPE tumor cDNAs, *TrkAIII* was detected in all 5 samples and ranged from ≈62 copies in sample 5F to ≈116 copies in sample 3F (Figure 7c). TaqMan qRT-PCR detected *TrkAIII* in two FFPE samples (2F and 5F), negative for TrkAIII expression by semi-quantitative RT-PCR, confirming enhanced sensitivity in *TrkAIII* detection, and no false negatives were detected, confirming the suitability of this assay for detecting *TrkAIII* in clinical samples.

### 3.3. Normalization of TrkAIII to β-actin Expression in TaqMan qRT-PCRs

To illustrate and highlight the importance of normalizing *TrkAIII* expression in comparative TaqMan qRT-PCR reactions, *TrkAIII* Ct values in pcDNA SH-SY5Y, TrkA SH-SY5Y, and TrkAIII SH-SY5Y cDNAs were normalized for *β-actin* expression [37,39]. Normalized *TrkAIII* expression in 25 ng of pcDNA SH-SY5Y controls, given the arbitrary value of 1, was 5.216-fold higher in 25 ng of TrkA SH-SY5Y cDNA and 2608.4-fold higher in 25 ng of TrkAIII SH-SY5Y cDNAs (Table 4). In addition, as *TrkAIII* was not detected in normal skin cDNA, comparisons of normalized *TrkAIII* expression in 25 ng of tumor 7B cDNA (low-level minor alternative *TrkAIII* splicing) and tumor 8B cDNA (high-level predominant alternative *TrkAIII* splicing), selected by semi-quantitative RT-PCR (Figure 7b), were made to 25 ng of normal skin cDNA spiked with 100 copies of purified *TrkAIII* PCR fragments. Compared with this control, given the arbitrary value of 1, *TrkAIII* expression in tumor 7B cDNA was 215.74-fold higher and in tumor 8B cDNA was 1313.16-fold higher (Table 4), which equated to ≈6-fold higher *TrkAIII* expression in tumor 8B compared with 7B. Notably, semi-quantitative RT-PCR barely detected *TrkAIII* expression in pcDNA or TrkA SH-SY5Y cDNAs, negating accurate comparisons.

The enhanced ability of TaqMan Assay 1 to detect *TrkAIII* mRNA, particularly in FFPE tumor RNAs, compared with semi-quantitative RT-PCR, supports the preferential use for this assay for the detection of *TrkAIII* mRNA expression in clinical samples.

## 4. Discussion

The real-time TaqMan-based qPCR assay described in this study provides a novel efficient, reproducible, specific, and sensitive method for detecting and quantifying *TrkAIII* mRNA expression in complex tumor cDNAs. Development of a TaqMan-based qRT-PCR approach to quantify *TrkAIII* expression followed the initial failure to accurately quantify *TrkAIII* expression in tumor RNAs by SYBR Green qRT-PCR, which we attribute to the high GC content (>60%) of the *TrkAIII* specific exon 5–8 splice junction target sequence [40], which led to the design of two potential TaqMan-based qRT-PCR strategies. The first strategy (Assay 1), in which *TrkAIII* specificity was provided by the TaqMan probe, exhibited optimal efficiency and specificity with high sensitivity, was able to detect as few as 10 *TrkAIII* copies in complex cDNAs, and was developed. The second strategy (Assay 2), in which *TrkAIII* specificity was provided by the reverse PCR primer, despite optimal efficiency and sensitivity in detecting *TrkAIII,* also consistently exhibited low-level amplification and detection of *fs-TrkA* and was discontinued. The reason for the elevated 103.398% amplification efficiency calculated for Assay 2 was not investigated, but it falls within the 90–105% range considered to represent high amplification efficiency (https://assets.fishersci.com/TFS-Assets/LSG/manuals/4387785c.pdf, accessed on 14 January 2025) and is likely to be attributable to the different primers and probes used in Assay 2.

The sensitivity of Assay 1 in detecting *TrkAIII* was superior to that of semi-quantitative RT-PCR; readily detected 10 copies of purified *TrkAIII*; was not compromised by increased cDNA complexity, as demonstrated by *TrkAIII* addition to *TrkAIII*-negative *Saccharomyces boulardii* (yeast) cDNA; and readily detected *TrkAIII* expression in tumor cell lines, fresh tumor tissue, and FFPE tumor tissue cDNAs, the latter being of importance for retrospective studies. With respect to the purification of RNA from FFPE tumor tissues, commercial kits are widely used, but they often vary in the quantity and quality of purified RNA obtained. The Thermo Fisher PureLink FFPE RNA isolation kit used in this study was chosen based upon previous experience in terms of RNA purity and quantity [25,27] and is supported by the recent confirmation that this particular kit is among the best in terms of RNA quality score and the percentage of RNA fragments > 200 nucleotides [41]. Collectively, these observations confirm the accuracy of this assay in comparative analyses of *TrkAIII* expression in clinical samples, which holds diagnostic, prognostic, and therapeutic relevance.

As detailed in the introduction, the development and validation of this *TrkAIII*-specific, highly sensitive TaqMan-based qRT-PCR method arises from semi-quantitative RT-PCR detection of *TrkAIII* in different tumor types, TrkAIII oncogenic activity and equivalence to a *TrkA*-fused oncogene in tumor models, expression promoted by conditions that characterize TMEs, inhibition by clinically approved Trk inhibitors, and recognition that TrkAIII is the only *TrkA* splice variant so far detected in tumor RNAs with oncogenic tyrosine kinase potential [16,17,20,21,25,26,27,28]. Collectively, these observations strongly suggest that oncogenic alternative *TrkAIII* splicing represents a frequent alternative to *TrkA-fused* oncogenes in different tumor types, which is consistent with the recognition that alternative splicing is an oncogene activation alternative to gene mutation in a wide variety of cancers, and in particular, tumors with low mutational burdens [30,31,32]. The actionable potential of TrkAIII, although mitigated by production via alternative splicing rather than gene mutation, is also supported by these observations and endows this assay with the potential to identify patients with therapy-resistant, advanced-stage, metastatic *Trk-fused* gene-negative tumors potentially driven by TrkAIII, for whom approval of third-line Trk inhibitor therapy could be extended.

## 5. Conclusions

We have successfully developed and validated a new, highly efficient, reproducible, and sensitive TaqMan-based qRT-PCR assay for the precise detection, quantification, and comparison of *TrkAIII* mRNA expression in complex RNAs extracted from either fresh or FFPE tumor tissues. When included in current precision oncology algorithms [33,34] and paired with *fs-TrkA* qRT-PCR and immunohistochemistry or phosphoproteomics to evaluate the level of alternative *TrkAIII* splicing and intracellular TrkA isoform(s) expression and activation, this assay will make it easier to identify patients with therapy-resistant, advanced-stage, metastatic *Trk-fused* gene-negative tumors potentially driven by TrkAIII, for whom approval of third-line effective Trk inhibitors could be extended.

## Figures and Tables

**Table 1:** Assay 1: Intra-assay and inter-assay reproducibility of purified TrkAIII cDNA.

Intra-Assay Ct and CV Values				Inter-Assay Ct and CV Values		
Copies	Mean Ct	SD	CV	Mean Ct	SD	CV
**10^7^**	17.965	0.035	0.002	18.775	0.732	0.039
**10^6^**	22.075	0.035	0.002	23.148	0.690	0.030
**10^5^**	26.280	0.061	0.002	26.620	0.378	0.014
**10^4^**	29.505	0.064	0.002	29.874	0.282	0.009
**10^3^**	33.240	0.127	0.004	33.943	0.559	0.016
**10^2^**	36.220	0.042	0.001	37.004	0.558	0.015
**10**	38.780	0.495	0.013	38.920	0.426	0.011

Ct = cycle threshold, SD = standard deviation, CV = coefficient of variation.

**Table 2:** Assay 1: Intra-assay and inter-assay reproducibility in detecting purified *TrkAIII* cDNA in *Saccharomyces boulardii* cDNAs.

Intra-Assay Ct and CV Values				Inter-Assay Ct and CV Values		
Copies	Mean Ct	SD	CV	Mean Ct	SD	CV
**10^7^**	17.231	0.133	0.008	17.295	0.138	0.008
**10^6^**	21.924	0.042	0.002	21.768	0.216	0.010
**10^5^**	25.046	0.085	0.003	25.159	0.176	0.007
**10^4^**	28.654	0.178	0.006	28.507	0.262	0.009
**10^3^**	31.866	0.165	0.005	31.977	0.222	0.007
**10^2^**	35.121	0.309	0.009	35.802	0.959	0.027
**10**	36.883	0.204	0.006	37.643	0.931	0.025

Ct = cycle threshold, SD = standard deviation, CV = coefficient of variation.

**Table 3:** Assay 1: Intra-assay and inter-assay reproducibility of detecting *TrkAIII* in purified TrkAIII SH-SY5Y cDNA.

Intra-Assay Ct Values				Inter-Assay Ct Values		
cDNA Input	Mean Ct	SD	CV	Mean Ct	SD	CV
**5 ng**	24.584	0.225	0.009	24.783	0.273	0.011
**0.5 ng**	28.293	0.345	0.012	29.010	0.833	0.029
**0.05 ng**	31.241	0.052	0.002	32.324	1.217	0.038
**0.005 ng**	34.866	0.244	0.007	35.305	0.667	0.019
**0.0005 ng**	38.535	0.215	0.006	39.167	0.634	0.016

Ct = cycle threshold, SD = standard deviation, CV = coefficient of variation.

**Table 4:** Fold change in *TrkAIII* expression normalized for β-actin expression.

Sample	Fold Change	Standard Deviation
pcDNA SH-SY5Y	1	0.16
*fs-*TrkA SH-SY5Y	5.216	0.113
TrkAIII SH-SY5Y	2608.4	0.084
N. Skin +100 TrkAIII copies	1	0.43
Tumor sample 7B	215.74	0.15
Tumor sample 8B	1313.16	0.14

## Data Availability

Data presented in this study are available from the corresponding author upon reasonable request.

## Supplementary Materials

The following supporting information can be downloaded at: https://www.mdpi.com/article/10.3390/cancers17030471/s1, Table S1: Details of the 11 fresh and 5 FFPE cutaneous malignant melanoma samples (CMMs) used in the development and validation of the *TrkAIII*-specific TaqMan-based qRT-PCR assay including tumor type, whether primary or metastatic; tumor stage; and reference to previously published patient numbers for these samples [27]. Figure S1: (a) Sanger sequence data printout of the *TrkAIII* cDNA region corresponding to the qRT-PCR amplicon in Assay 1 and (b) Nucleotide Blast of the sequence.

## References

[1] Martin-Zanca D., Hughes S.H., Barbacid M. (1986). A human oncogene formed by the fusion of truncated tropomyosin and protein tyrosine kinase sequences. Nature.

[2] Greco A., Roccato E., Pierotti M.A. (2004). TRK oncogenes in papillary thyroid carcinoma. Cancer Treat. Res..

[3] Martin-Zanca D., Oskam R., Mitra G., Copeland T., Barbacid M. (1989). Molecular and biochemical characterization of the human trk proto-oncogene. Mol. Cell. Biol..

[4] Klein R., Jing S.Q., Nanduri V., O’Rourke E., Barbacid M. (1991). The trk proto-oncogene encodes a receptor for nerve growth factor. Cell.

[5] Kaplan D.R., Hempstead B.L., Martin-Zanca D., Chao M.V., Parada L.F. (1991). The trk proto-oncogene product: A signal transducing receptor for nerve growth factor. Science.

[6] Hempstead B.L., Martin-Zanca D., Kaplan D.R., Parada L.F., Chao M.V. (1991). High-affinity NGF binding requires coexpression of the trk proto-oncogene and the low-affinity NGF receptor. Nature.

[7] Indo Y., Mardy S., Tsuruta M., Karim M.A., Matsuda I. (1997). Structure and organization of the human TRKA gene encoding a high affinity receptor for nerve growth factor. Jpn. J. Hum. Genet..

[8] Cocco E., Scaltriti M., Drilon A. (2018). NTRK fusion-positive cancers and TRK inhibitor therapy. Nat. Rev. Clin. Oncol..

[9] Iannantuono G.M., Riondino S., Sganga S., Rosenfeld R., Guerriero S., Carlucci M., Capotondi B., Torino F., Roselli M. (2022). NTRK Gene Fusions in Solid Tumors and TRK Inhibitors: A Systematic Review of Case Reports and Case Series. J. Pers. Med..

[10] Drilon A., Laetsch T.W., Kummar S., DuBois S.G., Lassen U.N., Demetri G.D., Nathenson M., Doebele R.C., Farago A.F., Pappo A.S. (2018). Efficacy of Larotrectinib in TRK Fusion-Positive Cancers in Adults and Children. N. Engl. J. Med..

[11] Liu D., Offin M., Harnicar S., Li B.T., Drilon A.E. (2018). Entrectinib: An orally available, selective tyrosine kinase inhibitor for the treatment of *NTRK*, *ROS1*, and *ALK* fusion-positive solid tumors. Ther. Clin. Risk Manag..

[12] Brose M.C., Westphalen B.C., Kehl K.L., Bernard-Gauthier V., Kurtinecz M., Brett R., Majdi A.A., Subbiah V., Pennell N.A., Drilon A.E. (2024). Outcomes of larotrectinib compared with real-world data from non-TRK inhibitor therapies in patients with TRK fusion cancer: VICTORIA study. J. Clin. Oncol..

[13] Desai A.V., Robinson G.W., Wu Y., Wang H., Basu E.M., Bagchi A., Casanova M., van Tilburg C.M., Campbell-Hewson Q., Armstrong A.E. (2024). Efficacy and safety of entrectinib in children with extracranial solid or primary central nervous system (CNS) tumors harboring NTRK or ROS1 fusions. J. Clin. Oncol..

[14] Roche. Entrectinib (Rozyltrek) U.S. Food and Drug Administration Website. https://www.accessdata.fda.gov/drugsatfda_docs/label/2019/212725s000lbl.pdf.

[15] Bayer. Larotrectinib (Vitrakvi) U.S. Food and Drug Administration Website. https://www.accessdata.fda.gov/drugsatfda_docs/label/2018/210861s000lbl.pdf.

[16] Tacconelli A., Farina A.R., Cappabianca L., Desantis G., Tessitore A., Vetuschi A., Sferra R., Rucci N., Argenti B., Screpanti I. (2004). TrkA alternative splicing: A regulated tumor-promoting switch in human neuroblastoma. Cancer Cell.

[17] Schramm A., Schowe B., Fielitz K., Heilmann M., Martin M., Marschall T., Köster J., Vandesompele J., Vermeulen J., de Preter K. (2012). Exon-level expression analyses identify MYCN and NTRK1 as major determinants of alternative exon usage and robustly predict primary neuroblastoma outcome. Br. J. Cancer.

[18] Arevalo J.C., Conde B., Hempstead B.L., Chao M.V., Martin-Zanca D., Perez P. (2000). TrkA immunoglobulin-like ligand binding domains inhibit spontaneous activation of the receptor. Mol. Cell. Biol..

[19] Watson F.L., Porcionatto M.A., Bhattacharyya A., Stiles C.D., Segal R.A. (1999). TrkA glycosylation regulates receptor localization and activity. J. Neurobiol..

[20] Farina A.R., Cappabianca L., Ruggeri P., Gneo L., Pellegrini C., Fargnoli M.C., Mackay A.R. (2018). The oncogenic neurotrophin receptor tropomyosin-related kinase variant, TrkAIII. J. Exp. Clin. Cancer Res..

[21] Farina A.R., Cappabianca L., Ruggeri P., Di Ianni N., Ragone M., Merolla M., Gulino A., Mackay A.R. (2012). Alternative TrkA Splicing and Neuroblastoma. Neuroblastoma—Present and Future.

[22] Ruggeri P., Farina A.R., Di Ianni N., Cappabianca L., Ragone M., Ianni G., Gulino A., Mackay A.R. (2014). The TrkAIII oncoprotein inhibits mitochondrial free radical ROS-induced death of SH-SY5Y neuroblastoma cells by augmenting SOD2 expression and activity at the mitochondria, within the context of a tumour stem cell-like phenotype. PLoS ONE.

[23] Farina A.R., Cappabianca L., Gneo L., Ruggeri P., Mackay A.R. (2017). TrkAIII signals endoplasmic reticulum stress to the mitochondria in neuroblastoma cells, resulting in glycolytic metabolic adaptation. Oncotarget.

[24] Cappabianca L., Ruggieri M., Sebastiano M., Sbaffone M., Martelli I., Ruggeri P., Di Padova M., Farina A.R., Mackay A.R. (2024). Molecular Characterization and Inhibition of a Novel Stress-Induced Mitochondrial Protecting Role for Misfolded TrkAIII in Human SH-SY5Y Neuroblastoma Cells. Int. J. Mol. Sci..

[25] Cappabianca L., Guadagni S., Maccarone R., Sebastiano M., Chiominto A., Farina A.R., Mackay A.R. (2019). A pilot study of alternative TrkAIII splicing in Merkel cell carcinoma: A potential oncogenic mechanism and novel therapeutic target. J. Exp. Clin. Cancer Res..

[26] Guadagni S., Farina A.R., Cappabianca L., Sebastiano M., Maccarone R., Zelli V., Clementi M., Chiominto A., Bruera G., Ricevuto E. (2020). Multidisciplinary Treatment, Including Locoregional Chemotherapy, for Merkel-Polyomavirus-Positive Merkel Cell Carcinomas: Perspectives for Patients Exhibiting Oncogenic Alternative Δ exon 6-7 TrkAIII Splicing of Neurotrophin Receptor Tropomyosin-Related Kinase A. Int. J. Mol. Sci..

[27] Cappabianca L., Zelli V., Pellegrini C., Sebastiano M., Maccarone R., Clementi M., Chiominto A., Ruggeri P., Cardelli L., Ruggieri M. (2023). The Alternative TrkAIII Splice Variant, a Targetable Oncogenic Participant in Human Cutaneous Malignant Melanoma. Cells.

[28] Sbaffone M., Jaffrain-Rea M.L., Cappabianca L., Carbonara F., Gianno F., Feola T., Ruggieri M., Zelli V., Maccarone R., Guadagni S. (2024). A study of alternative TrkA splicing identifies TrkAIII as a novel potentially targetable participant in PitNET progression. Biology.

[29] Treis D., Umapathy G., Fransson S., Guan J., Mendoza-García P., Siaw J.T., Wessman S., Gordon Murkes L., Stenman J.J.E., Djos A. (2022). Sustained Response to Entrectinib in an Infant With a Germline ALKAL2 Variant and Refractory Metastatic Neuroblastoma With Chromosomal 2p Gain and Anaplastic Lymphoma Kinase and Tropomyosin Receptor Kinase Activation. JCO Precis. Oncol..

[30] Siddaway R., Milos S., Vadivel A.K.A., Dobson T.H.W., Swaminathan J., Ryall S., Pajovic S., Patel P.G., Nazarian J., Becher O. (2022). Splicing is an alternate oncogenic pathway activation mechanism in glioma. Nat. Commun..

[31] Hwang W.L., Wolfson R.L., Niemierko A., Marcus K.J., DuBois S.G., Haas-Kogan D. (2019). Clinical Impact of Tumor Mutational Burden in Neuroblastoma. J. Natl. Cancer Inst..

[32] Castle J.C., Uduman M., Pabla S., Stein R.B., Buell J.S. (2019). Mutation-Derived Neoantigens for Cancer Immunotherapy. Front. Immunol..

[33] Lim K.H.T., Kong H.L., Chang K.T.E., Tan D.S.W., Tan I.B.H., Mohamad F., Soh S.Y., Pang B.N., Soo R.A., Choo S.P. (2022). Recommended testing algorithms for NTRK gene fusions in pediatric and selected adult cancers: Consensus of a Singapore Task Force. Asia Pac. J. Clin. Oncol..

[34] Nguyen M.A., Colebatch A.J., Van Beek D., Tierney G., Gupta R., Cooper W.A. (2023). NTRK fusions in solid tumours: What every pathologist needs to know. Pathology.

[35] Srinivasan M., Sedmak D., Jewell S. (2002). Effect of fixatives and tissue processing on the content and integrity of nucleic acids. Am. J. Pathol..

[36] Rogers-Broadway K.R., Karteris E. (2015). Amplification efficiency and thermal stability of rPCR instrumentation: Current landscape and future perspectives. Exp. Ther. Med..

[37] Ramezani A. (2021). CtNorm: Real time PCR cycle of threshold (Ct) normalization algorithm. J. Microbiol. Methods.

[38] Shechtman O., Doi S., Williams G. (2013). The Coefficient of Variation as an Index of Measurement Reliability. Methods of Clinical Epidemiology.

[39] Gur-Dedeoglu B., Konu O., Bozkurt B., Ergul G., Seckin S., Yulug I.G. (2009). Identification of endogenous reference genes for qRT-PCR analysis in normal matched breast tumor tissues. Oncol. Res..

[40] Mamedov T.G., Pienaar E., Whitney S.E., TerMaat J.R., Carvill G., Goliath R., Subramanian A., Viljoen H.J. (2008). A fundamental study of the PCR amplification of GC-rich DNA templates. Comput. Biol. Chem..

[41] Dube S., Al-Mannai S., Liu L., Tomei S., Hubrack S., Sherif S., Jabeen A., Ahmed E.I., Sanchez A., Mifsud W. (2025). Systemic comparison of quantity and quality of RNA recovered with commercial tissue extraction kits. J. Transl. Med..

