# Supplementary material for: A TaqMan-Based qRT-PCR Assay for Accurate Evaluation of the Oncogenic TrkAIII Splice Variant in Tumor cDNAs
Source: Cancers (Basel). 2025 Jan 30;17(3):471. doi: 10.3390/cancers17030471 (PMC11816089; doi:10.3390/cancers17030471)
Supplement: Supplementary file 1 [file cancers-17-00471-s001.zip › Supplementary Figure S1.pdf]

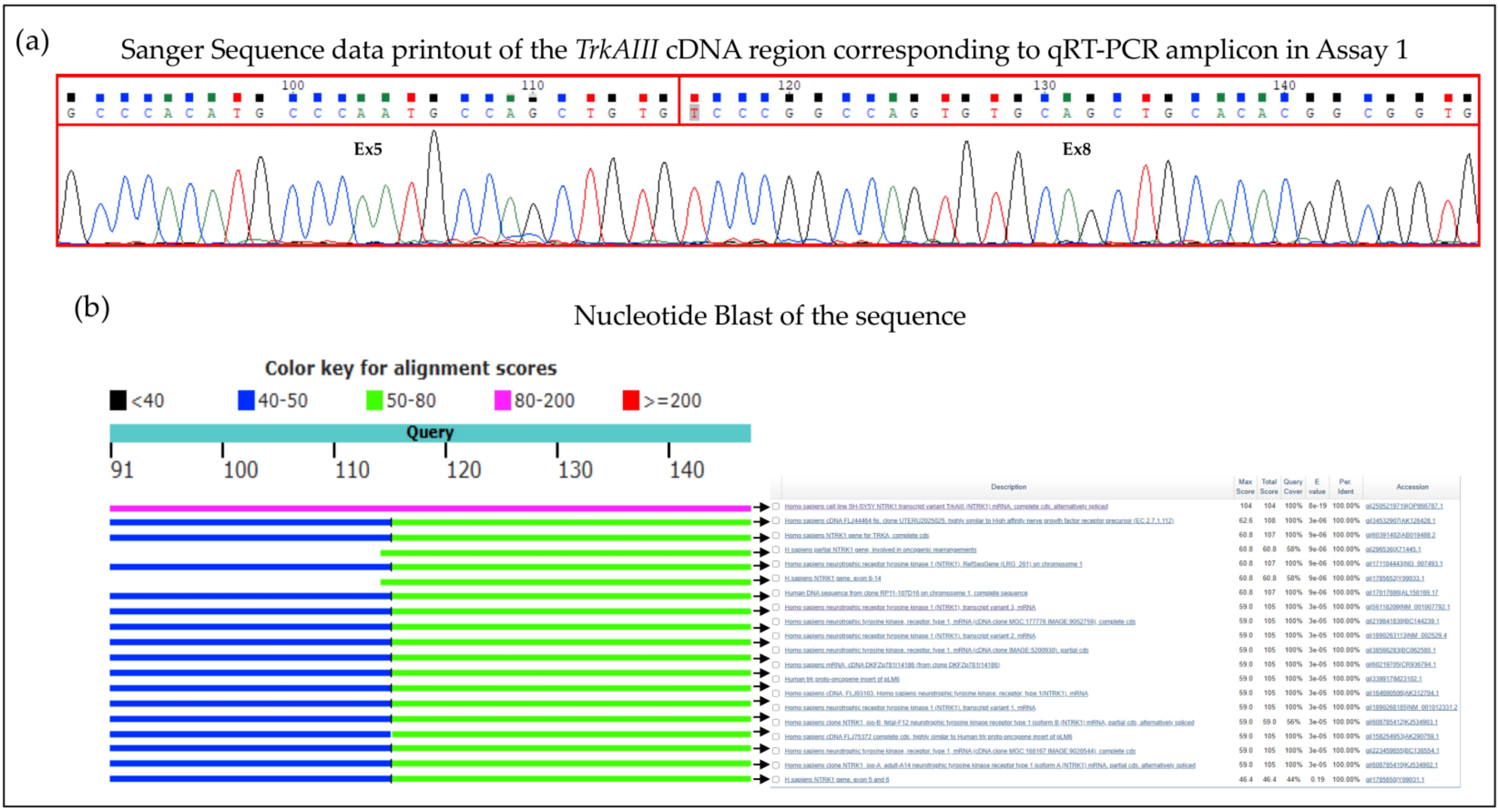

Figure S1. (a) Printout of the Sanger Sequence data for the *TrkAIII* cDNA region of interest corresponding to qRT-PCR amplicon in Assay 1 and (b) Nucleotide Blast of this sequence.
