# Supplementary material for: A TaqMan-Based qRT-PCR Assay for Accurate Evaluation of the Oncogenic TrkAIII Splice Variant in Tumor cDNAs
Source: Cancers (Basel). 2025 Jan 30;17(3):471. doi: 10.3390/cancers17030471 (PMC11816089; doi:10.3390/cancers17030471)
Supplement: Supplementary file 1 [file cancers-17-00471-s001.zip › Table S1-rev.pdf]

Table S1. Details of the 11 fresh and 5 FFPE cutaneous malignant melanoma samples (CMM) used in the development and validation of the *TrkAIII*-specific TaqMan-based qRT-PCR assay, including: tumor type, whether primary or metastatic, stage and previously published patient numbers for these samples in reference [27].

**Sample**	**tumor (stage)**	**in ref [25]**
1B	CMM metastasis (IIID)	p.20
2B	CMM metastasis (IIIC)	p.19
3B	CMM metastasis (IIIC)	p.15
4B	CMM primary (IIIC)	p.7
5B	CMM metastasis (IIID)	p.19
6B	CMM metastasis (IIID)	p.16
7B	CMM primary (1A)	p.5
8B	CMM metastasis (IIIC)	p.17
9B	CMM metastasis (IIIC)	p.22
10B	CMM metastasis (IIID)	p.24
11B	CMM metastasis (IV)	p.27
1F	CMM primary (III)	p.30a
2F	CMM metastasis (III)	p.30b
3F	CMM primary (IV)	p.28a
4F	CMM metastasis (IV)	p.28b
5F	CMM primary (III)	p.29a
